# Transcriptome analysis of SerpinB2-deficient breast tumors provides insight into deciphering SerpinB2-mediated roles in breast cancer progression

**DOI:** 10.1186/s12864-022-08704-4

**Published:** 2022-06-29

**Authors:** Yin Ji Piao, Hoe Suk Kim, Wonshik Han, Woo Kyung Moon

**Affiliations:** 1 Department of Biomedical Sciences, Seoul National University College of Medicine, 103 Daehak-ro, Jongno-gu, Seoul, 03080 Republic of Korea; 2 Department of Radiology, Sun Yat-Sen Memorial Hospital, Sun Yat-Sen University, No.107,Yanjiang Road West, Guangzhou, 510120 China; 3 Department of Radiology, Seoul National University Hospital and Seoul National University College of Medicine, 101 Daehak-ro, Jongno-gu, Seoul, 03080 Republic of Korea; 4 Cancer Research Institute, Seoul National University, 101 Daehak-ro, Jongno-gu, Seoul, 03080 Republic of Korea; 5 Biomedical Research Institute, Seoul National University Hospital, 101 Daehak-ro, Jongno-gu, Seoul, 03080 Republic of Korea; 6 Interdisciplinary Programs in Cancer Biology Major, Seoul National University Graduate School, 103 Daehak-ro, Jongno-gu, Seoul, 03080 Republic of Korea; 7 Integrated Major in Innovative Medical Science, Seoul National University Graduate School, 103 Daehak-ro, Jongno-gu, Seoul, 03080 Republic of Korea; 8 Department of Surgery, Seoul National University Hospital and Seoul National University College of Medicine, 101 Daehak-ro, Jongno-gu, Seoul, 03080 Republic of Korea

**Keywords:** RNA sequencing, SerpinB2-deficient mouse, MMTV-PyMT transgenic mouse, Breast cancer, Differentially expressed genes

## Abstract

**Background:**

SerpinB2 is highly expressed in immune and tumor cells and is involved in multiple biological functions, including cell survival and remodeling for disease progression. This study prepared SerpinB2-deficient mice and analyzed the differentially expressed genes (DEGs) to determine if loss of this protein delays mammary tumor progression.

**Results:**

A total of 305 DEGs (75 upregulated and 230 downregulated; > 1.5-fold difference, *P* < 0.05) were identified in SB2−/−;PyMT tumors compared with PyMT tumors. The DEGs were mainly involved in immune and inflammatory responses related to T cell differentiation, IFN-γ production, and lymphocyte chemotaxis based on 61 enriched GO terms, hierarchical clustering, KEGG pathways, and a functionally grouped annotation network. The significantly changed DEGs (Anxa3, Ccl17, Cxcl13, Cxcr3, IFN-γ, Nr4a1, and Sema3a) annotated with at least two GO categories in SB2−/−;PyMT tumors was validated by qRT-PCR.

**Conclusions:**

SerpinB2 deficiency alters the expression of multiple genes in mammary tumors, which might cause a delay in PyMT-induced mammary tumor progression.

**Supplementary Information:**

The online version contains supplementary material available at 10.1186/s12864-022-08704-4.

## Background

SerpinB2 or plasminogen activator inhibitor-2 (PAI-2) is an inhibitor of extracellular urokinase plasminogen activator (uPA) and is expressed in a number of cell types, especially tumor cells and immune cells [1]. SerpinB2 is involved in diverse cellular functions including cell survival, cell differentiation, inflammation, immunity, cell adhesion, migration, and extracellular matrix (ECM) remodeling in diseases including cancer by interacting with intracellular and extracellular proteins [2–4]. This implies that SerpinB2 is associated with tumorigenesis, invasion, and metastasis in the complex tumor system. SerpinB2 is a protumorigenic or antitumorigenic gene depending on the cancer type [5–7]. Mouse models of SerpinB2-deficient mammary tumors are useful for addressing various scientific questions regarding the in vivo functions of SerpinB2 during breast cancer progression.

We generated SerpinB2-deficient MMTV-PyMT (SB2−/−;PyMT) mice by intercrossing SerpinB2-deficient (SB2−/−) mice with C57BL/6 strain background MMTV-PyMT (PyMT) mice widely used to study human breast cancer. Mammary tumor onset and progression were significantly delayed in SB2−/−;PyMT mice compared with those in PyMT mice. We aimed to analyze transcriptome profiles and networks of mammary tumor tissue samples collected from age-matched PyMT and SB2−/−;PyMT mice using RNA-Sequencing (RNA-Seq) technology to understand the underlying mechanism by which SerpinB2 deficiency is involved in delayed breast cancer development and metastasis. Gene Ontology (GO) terms, pathway enrichment, and a functionally organized GO/pathway network of differentially expressed genes (DEGs; 1.5-fold change (FC) and *P* < 0.05) identified in SB2−/−;PyMT tumors relative to PyMT tumors were analyzed. This work provides insights into the biological functions and regulatory mechanisms of SerpinB2 in mammary tumors.

## Results

### SB2−/−;PyMT mice exhibit delayed onset and growth of mammary tumors

**Fig. 1:**
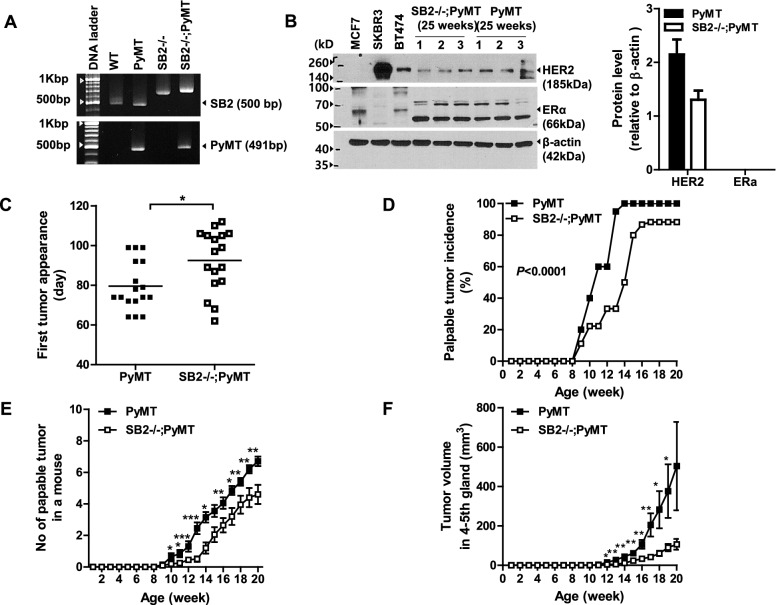
SerpinB2 deficiency delays the appearance of the first tumor, reduces the palpable tumor incidence and results in fewer palpable tumors and reduced tumor volume. (**A**) Representative genotyping of SerpinB2 and PyMT using genomic DNA isolated from the tail samples of wild-type, PyMT, SB2−/− and SB2−/−;PyMT mice. (**B**) Western blot analysis of ERα and HER2 in MCF 7, SKBR3, and BT474 cells and the tumor lysates of PyMT and SB2−/−;PyMT mice The data are expressed as means ± standard errors from the primary tumors of 3 mice per group. (**C**) Average time (day) for the appearance of the first palpable tumor in PyMT and SB2−/−;PyMT mice. (**D**) Percentage of the mice with palpable tumors in PyMT and SB2−/−;PyMT mice by 20 weeks of age after birth. (**E**) Average number of multifocal palpable tumors in PyMT and SB2−/−;PyMT mice at 8–20 weeks of age. (**F**) Tumor volumes in the 4th–5th gland of PyMT and SB2−/−;PyMT mice at 8–20 weeks of age. The time-to-first appearance and multifocal numbers of a palpable tumor per mouse are expressed as means ± standard errors from 20 mice per group. *p < 0.05, **0.001 < *p* < 0.05

To investigate the potential in vivo role of SerpinB2 in breast cancer development and progression, SerpinB2-deficient PyMT (SB2−/−;PyMT) mice were produced by crossing SB2−/− female mice with C57BL/6 strain background PyMT males, and the PyMT transgene and SerpinB2-deficient genotype in all female offspring were analyzed by PCR (Fig. 1A). The protein expression level of ERα was not detected in 25-week-old SB2−/−;PyMT and PyMT mice tumors, while the HER2 protein level was comparably lower in SB2−/−;PyMT tumors (1.31 ± 0.17) than in PyMT tumors (2.15 ± 0.28), suggesting that SerpinB2 deficiency may be associated with reduced HER2 expression status (Fig. 1B). The raw data of Fig. 1 A, B are shown in Supplementary data 1. The time-to-first appearance of a palpable tumor per mouse (mean ± standard error), number of palpable tumor-bearing mice per group (%), as well as multifocal tumor numbers per mouse per time point (mean ± standard error) were investigated. The first appearance of palpable tumors in PyMT mice and SB2−/−;PyMT mice was observed at 79.53 ± 2.98 days and 92.47 ± 3.75 days after birth, respectively (*P* = 0.011, Fig. 1 C). Palpable mammary tumors developed within 9–14 weeks of birth in 20–100% of PyMT mice and 11–50% of SB2−/−;PyMT mice. At 20 weeks of age, 100% of the PyMT mice and 88.24% of the SB2−/−;PyMT mice had palpable mammary tumors (*P* < 0.0001, Fig. 1 D). Prior to 12 weeks of age, multifocal tumors arose more slowly in the mammary glands of SB2−/−;PyMT mice than in those of PyMT mice, and the number of multifocal palpable primary tumors in PyMT mice and SB2−/−;PyMT mice at 20 weeks of age were 7.05 ± 0.34 and 4.6 ± 0.60, respectively (Fig. 1E). Among 10 mammary glands, SB2−/−;PyMT mice exhibited a remarkably slower tumor growth rate in 4th and 5th mammary glands, and the volume of tumors generated from the 4th and 5th mammary glands of SB2−/−;PyMT mice was smaller at 20 weeks compared with those of PyMT mice (Fig.1 F).

### Identification of DEGs between SB2−/−;PyMT and PyMT

**Fig. 2:**
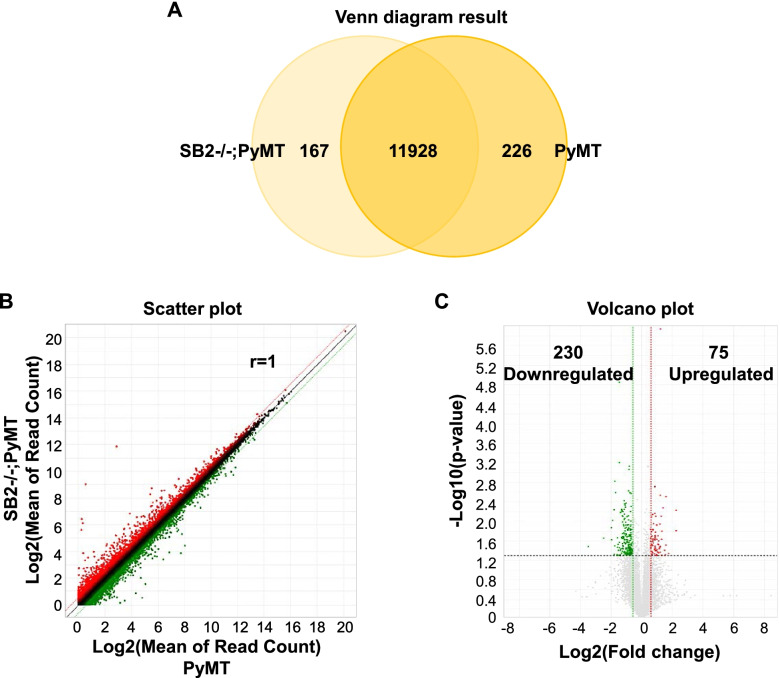
Analysis of differentially expressed genes (DEGs) between SB2−/−;PyMT and PyMT tumors determined by RNA-Seq (**A**) Venn diagram of the gene expression level in the two groups. (**B**) Scatter plot of global expression between samples; the Pearson correlation coefficient is shown. (**C**) Volcano plot of DEGs in the two groups. Red, green, and black dots represent genes that are significantly upregulated, significantly downregulated, and not significantly different, respectively

The Venn diagram shows that 11,928 genes overlapped between SB2−/−;PyMT and PyMT tumors. Genes with expression changes at an average of normalized RC (log2) > 5 in both groups showed that 167 and 226 genes did not overlap in SB2−/−;PyMT tumors and PyMT tumors, respectively. (Fig. 2A). DEGs between SB2−/−;PyMT and PyMT tumors were selected by ranking genes with a log10 *P* value < 0.05 and plotting against the log2 (FC) in scatter and volcano plots (Fig. 2B, C). We identified 305 DEGs including 75 upregulated (FC ≥ 1.5) and 230 downregulated genes (FC ≤ 1.5) in SB2−/−;PyMT tumors (Supplementary Table 1) and the largest variations in DEGs are shown in Tables 1 and 2.

**Table 1:** Top 10 upregulated mRNAs in SB2−/−;PyMT tumors compared to PyMT tumors

Entrez ID	Gene symbol	Description	Fold change	*P* value
654	SerpinB8	Serine or cysteine peptidase inhibitor, clade B (ovalbumin), member 8	4.771	0.016
650	SerpinB11	Serine (or cysteine) peptidase inhibitor, clade B (ovalbumin), member 11	4.758	0.006
690	Gpr39	G protein-coupled receptor 39	3.014	0.003
17,492	Clec2f	C-type lectin domain family 2, member f	2.909	0.049
10,729	St8sia6	ST8 alpha-N-acetyl-neuraminide alpha-2,8-sialyltransferase 6	2.661	0.005
1378	Slc35d3	Solute carrier family 35, member D3	2.457	0.050
643	SerpinB5	Serine (or cysteine) peptidase inhibitor, clade B (ovalbumin), member 5	2.352	0.000
620	Panct2	Pluripotency-associated noncoding transcript 2	2.324	0.003
13,538	Col25a1	Collagen type XXV alpha 1	2.216	0.036
6348	Fam167a	Family with sequence similarity 167, member A	2.172	0.031

**Table 2:** Top 10 downregulated mRNAs in SB2−/−;PyMT tumors compared to PyMT tumors

Entrez ID	Gene symbol	Description	Fold change	*P* value
657	Cdh19	Cadherin 19	0.089	0.033
15,652	Amtn	Amelotin	0.178	0.022
14,992	Car6	Carbonic anhydrase 6	0.253	0.014
19,606	Dmbt1	Deleted in malignant brain tumors 1	0.256	0.024
19,845	Retn	Resistin	0.289	0.048
15,727	Cxcl13	C-X-C motif chemokine ligand 13	0.332	0.034
4882	Akr1c14	Aldo-keto reductase family 1 member 14	0.347	0.040
14,217	Elavl2	ELAV like RNA binding protein 2	0.356	0.050
607	D2hgdh	D-2-hydroxyglutarate dehydrogenase	0.363	0.000
16,113	Tmem132c	Transmembrane protein 132C	0.363	0.035

### GO enrichment analysis

**Fig. 3:**
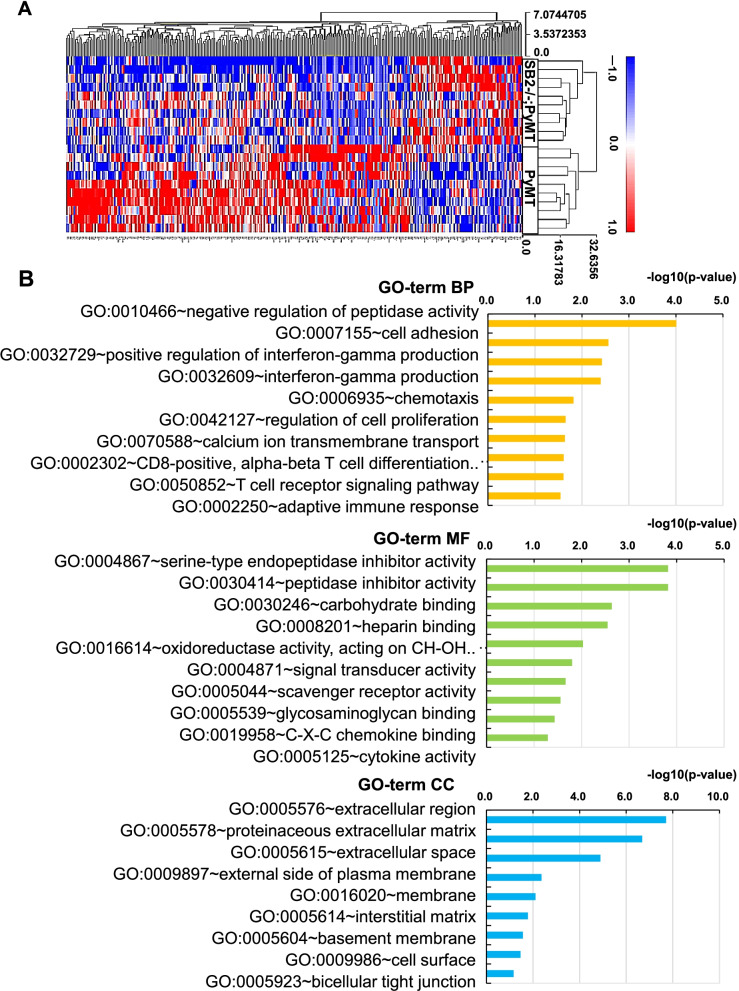
Hierarchical clustering of DEGs and GO enrichment analysis. (**A**) Gene expression heatmap based on hierarchical clustering in two groups. (**B**) Histogram of the top 10 enriched GO terms obtained from 305 DEGs. Yellow: biological process (BP); green: molecular function (MF); blue: cellular component (CC)

Hierarchical clustering of 75 upregulated and 230 downregulated DEGs in SB2−/−;PyMT tumors compared to PyMT tumors (FC ≥ 1.5, FC ≤ 1.5, *P* < 0.05) (Fig. 3A) was followed by GO enrichment analysis. DEGs were enriched in the following GO terms: biological processes (BP; 34), molecular function (MF; 18), and cellular compartment (CC; 9) (Supplementary Table 2), and the top ten subclasses of GO enrichment terms are shown in Fig. 3B.

### Pathway enrichment analysis

The DEGs were mapped to three terms in the Kyoto Encyclopedia of Genes and Genomes **(**KEGG) database (http://www.genome.ad.jp/kegg/) [5–7]; cytokine-cytokine receptor interaction pathways, neuroactive ligand-receptor interaction, and pancreatic secretion (Table 3).

**Table 3:** KEGG pathway enrichment in SB2−/−;PyMT tumors based on GO terms from DEGs

Term	Count	Percentage of DEGs	*P* value	Log10 (*P* value)	Genes
mmu04060: Cytokine-cytokine receptor interaction	9	0.03	0.005	2.261	Lep, Il12rb1, Cxcl13, Cxcr6, Cxcl2, IFN-γ, Tnfsf14, Cxcr3, Ccl7
mmu04080: Neuroactive ligand-receptor interaction	9	0.03	0.014	1.853	Lep, Avpr2, P2rx3, P2ry1, Mc2r, Htr4, Adra1b, Htr1d, Sctr
mmu04972: Pancreatic secretion	5	0.02	0.025	1.595	Pla2g1b, Cpa2, Prkcg, Pla2g2d, Sctr

### Functionally grouped annotation network

**Fig. 4:**
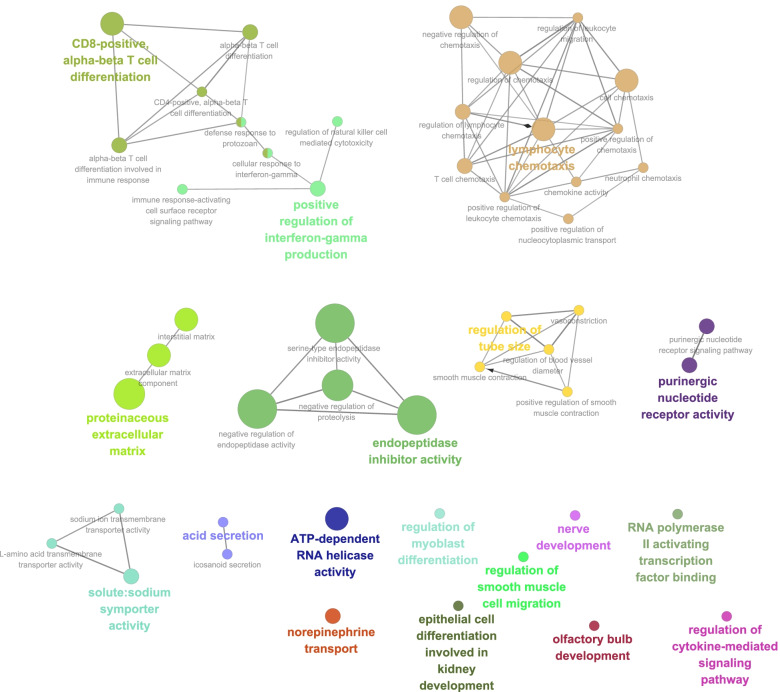
Grouping of network based enriched GO terms. A functionally grouped network was visualized using Cytoscape software version 2.6.2 with the ClueGO plugin

Cytoscape software functionally organized the GO network for DEGs in SB2−/−;PyMT tumors. Overall, 51 GO terms in the BP, MF, and CC categories were significantly enriched, and these categories were organized into 18 annotation networks that reflected the relationships between the terms based on the integration of their associated genes (Fig. 4). The main networks were endopeptidase inhibitor activity, lymphocyte chemotaxis, proteinaceous extracellular matrix, CD8-positive α-β T-cell differentiation, and positive regulation of interferon-γ production.

### Selection of genes associated with the 9 functional GO categories

**Fig. 5:**
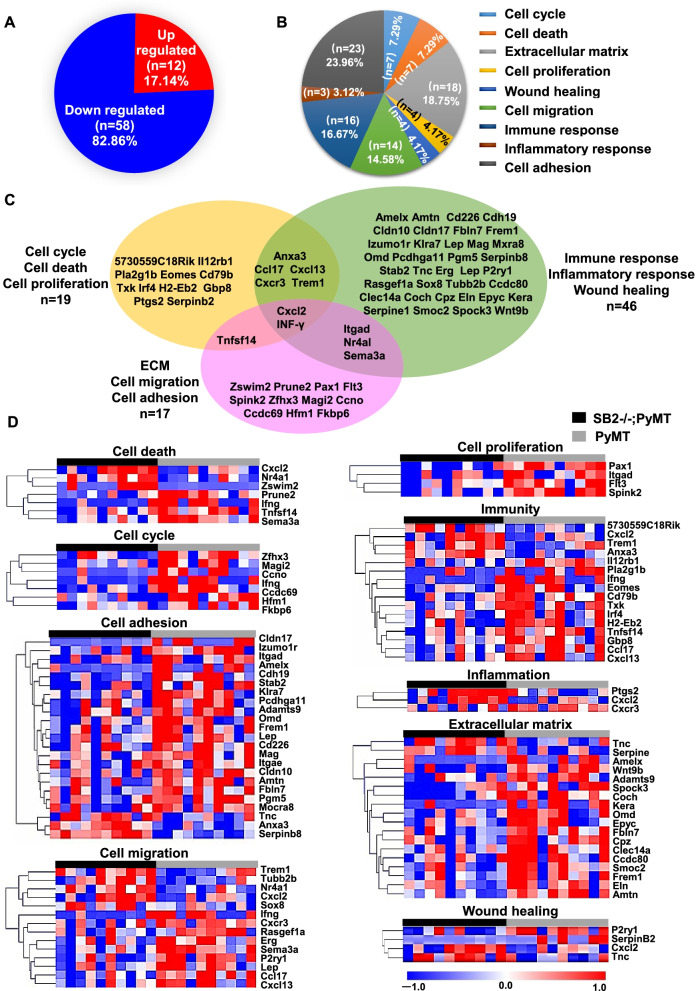
Extraction of genes annotated with at least two GO functional categories. (A-C) Pie charts of the genes annotated with nine functional categories stated in the legend of panel. (D) Gene expression heatmap based on hierarchical clustering of the genes annotated with nine functional categories

Finally, we selected 82 genes belonging to nine functional categories (cell cycle, cell proliferation, cell death, cell migration, cell adhesion, wound healing, ECM, immunity, and inflammation) from 305 DEGs identified in SB2−/−;PyMT tumors. Overall, 12 of these DEGs (17.14%) were downregulated and 58 (82.86%) were upregulated (Fig. 5A). These genes were involved in the cell cycle (7.29%), cell death (7.29%), ECM modification (18.75%), cell proliferation (4.17%), wound healing (4.17%), cell migration (14.58%), immunity (16.67%), inflammation (3.12%), and cell adhesion (23.96%) (Fig. 5B). Several DEGs were annotated with at least two GO categories (Fig. 5C and Table 4). Hierarchical clustering and detailed information on these genes belonging to the nine functional categories are shown in Fig. 5D and Supplementary Table 3.

**Table 4:** DEGs annotated with at least two functional categories

Gene symbol	Description	Fold change	*P* value
Anxa3	Annexin A3	1.58	0.008
Ccl17	Chemokine (C-C motif) ligand 17	0.413	0.008
Cxcl2	Chemokine (C-X-C motif) ligand 2	1.957	0.048
Cxcl13	Chemokine (C-X-C motif) ligand 13	0.332	0.034
Cxcr3	Chemokine (C-X-C motif) receptor 3	0.608	0.047
IFN-γ	Interferon gamma	0.575	0.038
Itgad	Integrin, alpha D	0.522	0.045
Nr4a1	Nuclear receptor subfamily 4, Group A, member 1	2.019	0.038
Sema3a	Semaphorin 3A	0.455	0.004
Tnfsf14	Tumor necrosis factor (ligand) superfamily, member 14	0.63	0.043
Trem1	Triggering receptor expressed on myeloid cells 1	1.856	0.039

### Quantitative real-time RT-PCR (qRT-PCR) analyses of DEGs annotated with at least two functional categories

**Fig. 6:**
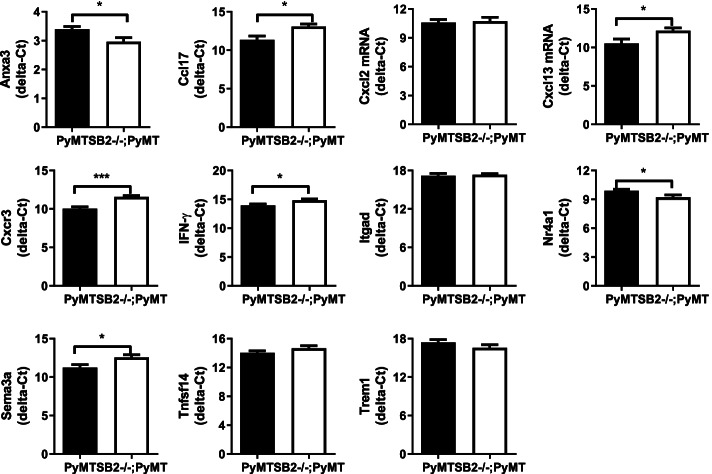
Relative expression analysis of DEGs annotated with at least two functional categories by qRT-qPCR. The expression levels of these genes in individual tumor samples of PyMT mice (black bars) and SB2−/−;PyMT mice (white bars) were normalized by γ-actin. The relative mRNA levels were ∆Ct, which is the difference in Ct values for genes of interest and internal reference gene γ-actin

Anxa3, Ccl17, Cxcl13, Cxcr3, INF-γ, Nr4a1, and Sema3a mRNA levels were significantly changed in SB2−/−;PyMT tumors compared with PyMT tumors. The gene of Anxa3 and Nr4a1 ∆Ct values which normalized with the γ-actin housekeeping gene were significantly decreased (Anxa3: 3.394 ± 0.094 vs. 2.961 ± 0.142, *P* = 0.025; Nr4a1: 9.898 ± 0.133 vs. 9.213 ± 0.245 *P* = 0.028), which means that the mRNA levels of Anxa3 and Nr4a1 were significantly increased in SB2−/−;PyMT tumors compared with PyMT tumors. The ∆Ct values of Ccl17, Cxcl13, Cxcr3, INF-γ, and Sema3a were significantly increased in SB2−/−;PyMT tumors compared with PyMT tumors (Ccl17: 11.35 ± 0.509 vs. 13.08 ± 0.324 *P* = 0.01; Cxcl13: 10.51 ± 0.591 vs. 12.18 ± 0.368 *P* = 0.028; Cxcr3: 10.04 ± 0.222 vs. 11.56 ± 0.169 *P* = 0.0003; INF-γ: 13.94 ± 0.266 vs. 14.85 ± 0.217 *P* = 0.024; Sema3a: 11.24 ± 0.381 vs. 12.56 ± 0.348 *P* = 0.021; Tnfsf14: 13.16 ± 0.385 vs. 14.67 ± 0.336 *P* = 0.0124). While no significant differences were noted in Cxcl2, Itgad, Tnfsf14, and Trem1 (Cxcl2: 10.59 ± 0.314 vs. 10.73 ± 0.409 *P* = 0.787; Itgad: 17.18 ± 0.359 vs. 17.32 ± 0.181 *P* = 0.735; Tnfsf14: 14.67 ± 0.336 vs. 14.03 ± 0.262 *P* = 0.159; Trem1: 17.40 ± 0.443 vs. 16.56 ± 0.489 *P* = 0.221) (Fig. 6).

## Discussion

SerpinB2 is expressed by a variety of cells and is implicated in various diseases including cancer, inflammation, and immune-associated diseases [8]. Dougherty et al. produced SerpinB2-deficient (SB2−/−) mice to examine the potential in vivo functions of SerpinB2 in murine development [9]. MMTV-PyMT transgenic (PyMT) mice are widely used for studying breast cancer development and metastasis, especially the luminal B subtype [10]. There is a lack of studies analyzing the in vivo role of SerpinB2 in transgenic and knockout animal models of breast cancer. We generated SerpinB2-deficient MMTV-PyMT (SB2−/−;PyMT) mice by crossing SB2−/− mice with PyMT mice to provide insights into the in vivo function of SerpinB2 in mammary tumor development and progression and found that SerpinB2 deficiency resulted in a delay in PyMT-induced mammary tumor initiation and progression. RNA-Seq is a valuable method for quantifying transcriptome signatures and deciphering the regulatory network underlying breast tumor progression from primary tumor development to metastasis [11, 12]. Transcriptome profiling using RNA-Seq was performed in mammary tumors of PyMT and SB2−/−;PyMT mice to obtain the DEGs, the genetic network, and understand how SerpinB2 deficiency affects delayed breast cancer development and metastasis.

Three hundred and five DEGs were observed in SB2−/−;PyMT tumors, including 75 upregulated genes and 230 downregulated genes compared with PyMT tumors. SerpinB2 subfamily members (SerpinB1–13) inhibit activity of cytotoxic and apoptotic proteases and papain-like enzymes and non-inhibitory molecules [13]. SerpinB8, SerpinB11, and SerpinB5 were among the top 10 upregulated DEGs in SB2−/−;PyMT tumors. Particularly, SerpinB5 (maspin) is a tumor suppressor that inhibits metastasis in human mammary epithelial cells [14]. Cdh19 (cadherin 19) was one of the top 10 downregulated DEGs identified in SB2−/−;PyMT tumors; however, there are no reports on the role of this gene in adherent junction formation associated with breast cancer progression. It is important to investigate whether Cdh19 can induce invasion or dissemination in the mammary epithelium. Amtn (amelotin) may play a role in the controlled mineralization of hydroxyapatite that is an early diagnostic marker for breast cancer, is involved in enhancing breast cancer progression, and is associated with poor breast cancer prognosis [15]. Therefore, upregulated SerpinB5 and downregulated Amtn may be involved in suppressing mammary cancer development and lymph node metastasis in SB2−/−;PyMT mice.

The role of SerpinB2 expression by diverse types of cells in cancer growth and metastasis is controversial. High SerpinB2 expression is noted in mouse melanoma cell line of B16 cells, and human melanoma cell line of M24met inhibiting metastasis [16, 17]. In addition, perivascular expression of SerpinB2 in C8161 human melanoma cells promotes brain metastasis [18]. The SerpinB2-deficient human lung cancer cell line H2030-BrM3 and human breast cancer cell line MDA231-BrM2 do not form brain metastases because the deleterious effects of SerpinB2 on the vascular attachment and survival of cancer cells are inhibited [19]. Recently, Jin et al. showed that SerpinB2 knockdown in MDA-MB-231 breast cancer cells suppressed their migratory activity and lung metastasis [20]. SerpinB2-deficient mouse embryonic fibroblasts exhibit increased pancreatic tumor growth and local invasion with reduced collagen deposition [4], whereas fibroblasts overexpressing SerpinB2 reduce human breast tumor cell apoptosis. MDA-MB-231 promotes breast tumor growth and lung metastasis and reduces tumor apoptosis [21]. SerpinB2 upregulation in monocytes/macrophages following infection or stimulation with inflammatory mediators is involved in a cellular response to delay cell death, possibly allowing cells to complete vital functions such as lymphocyte activation and antigen presentation [22]. SerpinB2-deficient mice produce more IgG2c and OVA-specific IFN-γ-secreting T cells than wild-type mice suggesting that SerpinB2 regulates adaptive immunity [3]. In the aforementioned studies [19, 20], SerpinB2 expression substantially mediates physiological and pathological functions including inhibitory activity against serine protease plasminogen activators, ECM remodeling, cell survival and migration, inflammation, and immunity. Thus, the role of SerpinB2 in cancer development and aggressive progression may depend on the cancer type.

Our results showed that SerpinB2 plays roles in immunity, inflammation, chemotaxis, and ECM modulation. GO enrichment and functional network analysis of DEGs identified from SB2−/−;PyMT tumors revealed multiple biological processes including T cell differentiation, INF-γ production, lymphocyte chemotaxis, ECM, and peptidase inhibitor activity which is consistent with previous studies [2, 3, 13, 23, 24]. Eleven DEGs (Anxa3, Ccl17, Cxcl2, Cxcl13, Cxcr3, INF-γ, Itgad, Nr4a1 Sema3a, Tnfsf14, and Trem1) were annotated with at least two GO categories. We here validated quantitative expression levels of these genes between PyMT and SB2−/−;PyMT tumors using qRT-PCR. Seven of the 11 genes (Anxa3, Ccl17, Cxcl13, Cxcr3, IFN-γ, Nr4a1, and Sema3a) exhibited identical RNA-Seq and qRT-PCR results, whereas four genes (Cxcl2, Itgad, Tnfsf14, and Trem1) did not. Thus, the RNA-Seq and qRT-PCR findings agreed on 63.6% of the genes. RNA-Seq is a strong tool, however variations in starting quantities or cDNA quality can obscure quantitative differences between two samples [25]. As a result, more precise methods are needed to confirm the exact copy counts of specific mRNAs. The only approach to avoid this error is to use internal standard-normalized qRT-PCR.

Gene of Ccl17, Cxcl13, Cxcr3, IFN-γ, and Sema3a, were significantly decreased while the Anxa3 and Nr4a1 were significantly upregulated in SB2−/−;PyMT mammary tumors. High levels of Ccl17, Cxcl13, and Cxcr3 in mammary tumors or tumor-associated macrophages induce cancer cell migration which is associated with metastasis disease [26–29]. Ccl17 and Cxcr3 increased in mammary tumors that protects cancer cell survival and induce tumor growth to promote breast cancer lung metastasis [28, 29] Hypoxia-induced chemokine Sema3a stimulates tumor-associated macrophages to the alternative type (M2), leading to pro-tumor immunity [30]. And the tumor suppresser gene of Nr4a1 in TNBC was decreased cancer cell proliferation and invasiveness [31]. These studies support our findings that low levels of Ccl17 and Cxcl13, Cxcr3, and Sema3a as well as high levels of Nr4a1 in SB2−/−;PyMT tumors may delay mammary tumor development and may lead to decreased metastasis. However, in SB2−/−;PyMT tumors, a high levels of IFN-γ, which activates the antitumor immune response [32], and a low levels of Anxa3, which controls cancer cell invasion and lung metastasis [33], do not support SerpinB2 deficiency causing a delay in PyMT-induced mammary tumor progression. Given these results, the crosstalk between the immune system and cancer cells mediated by these cytokines and chemokines in SB2−/−;PyMT tumors during tumor initiation and progression in breast cancer requires further study.

## Conclusion

SerpinB2-deficient MMTV-PyMT mice (SB2−/−;PyMT) exhibited altered expression of multiple genes related to many aspects of immunity, inflammation, chemotaxis, cell adhesion, ECM modulation, peptidase activity, and cell proliferation suggesting that they delay PyMT-induced mammary cancer development and metastasis. Further studies are required to understand the complex roles of multiple genes identified in our study in tumor microenvironments.

## Methods

### Animals

PyMT mice originally generated by Bill Muller’s laboratory [10] on a C57BL/6 background were kindly provided by Dr. Sandra Gendler (Mayo Clinic, Scottsdale, AZ, USA). SerpinB2-deficient (B6.129S1-Serpinb2tm1Dgi/J) (SB2−/−) mice were provided by Jackson Laboratory (Bar Harbor, ME, USA). Male PyMT mice were mated with female SB2−/− mice to generate progeny with the SB2−/− or SB2+/− genotype. Female SB2+/− and male SB2+/−;PyMT mice were mated against each other to generate SB2−/−;PyMT female littermates. The mice used in the RNA-Seq experiment were age-matched PyMT and SB2−/−;PyMT females (20–25 weeks). Animal care and experimental procedures were conducted according to the guidelines on the ethical use of animals. The study protocol was approved by the Institutional Animal Care and Use Committee of Seoul National University (SNU-150210-3-4). All methods are reported in accordance with ARRIVE guidelines (https://arriveguidelines.org) for the reporting of animal experiments.

### Genotyping

Chromosomal DNA was isolated from a 2-mm piece of the tail tip of mice. Mice were genotyped for the PyMT allele by polymerase chain reaction (PCR) using previously described primers [34]: forward primer, 5′-AGTCACTGCTACTGCACCCAG-3′; reverse primer, 5′-CTCTCCTCAGTTCTTCGCTCC-3′. The genotypes of SB2−/− mice were confirmed by additional multiplex PCR using previously described primers [9]: 5 (SerpinB2 exon 8 5′-TTTGATAGGCGGGTTGTTTCTCTGT-3′), 6 (neospecific 5′-CAGCCGAACTGTTCGCCAGG-3′), and 7 (3 sequence flanking SerpinB2 5′-GTTTGTCCACCATGCTCCCTCTA-3′). Primers 5 and 7 amplified a 500-bp product from the endogenous SerpinB2 allele, and primers 6 and 7 amplified a 650-bp product from the targeted allele.

### RNA extraction from tumor tissues

Mammary tumor tissues were collected from age-matched SB2−/−;PyMT (*n* = 10) and PyMT (n = 10) mice (20 weeks old [*n* = 2], 22 weeks old [n = 2], and 25 weeks old [*n* = 6] per group). Total RNA content was extracted from a 20-mg piece of the tumor using TRIzol reagent (Invitrogen, Carlsbad, CA, USA) according to the manufacturer’s instructions. RNA concentration was measured using a NanoDrop 2000 spectrophotometer (Thermo Fisher Scientific, Waltham, MA, USA). RNA integrity number (RIN) was determined using an Agilent RNA 6000 Nano kit following the manufacturer’s protocol on an Agilent 2100 bioanalyzer (Agilent, Santa Clara, CA, USA).

### RNA-Seq and DEG analysis

Sequencing libraries were constructed using the QuantSeq 3′ mRNA-Seq library prep kit (Lexogen, South Morang Victoria, Austria) according to the manufacturer’s instructions. High-throughput RNA-Seq was performed for 75 single-end sequences using the NextSeq 500 system (Illumina, San Diego, CA, USA). DEGs between SB2−/−;PyMT, and PyMT tumors were determined based on counts from unique and multiple alignments using coverage in BEDTools [35]. The read count data were processed based on the quantile normalization method using EdgeR within R (R Development Core Team, 2016) by Bioconductor [36]. Genes with a *P* value < 0.05 were ranked by the log10 *P* value and plotted against the log2 (FC) in a “volcano” plot. Genes that were upregulated and downregulated with a *P* value < 0.05 and log ratio >1.5 were considered DEGs.

### GO, KEGG pathway, enrichment, and functional network analysis

Biological functional categories enriched in DEGs were identified using the functional annotation and clustering tool of the Database for Annotation, Visualization, and Integrated Discovery v6.7 (https://david.ncifcrf.gov/) [37–39]. Significant GO terms were identified after multiple testing adjustments using the Benjamini–Hochberg method; Benjamini < 0.05, which indicated a statistically significant difference and GO term lists BP, MF, and CC were matched [40]. KEGG pathway enrichment analyses was performed using the KEGG pathway software (https://www.kegg.jp/kegg/) from the Kanehisa laboratory and the threshold value was set at *P* < 0.05. The functionally organized GO/pathway network was created using Cytoscape software version 2.6.2 (http://www.cytoscape.org/) with the ClueGO plugin (http://www.ici.upmc.fr/cluego/cluegoDownload.shtml) [41].

### qRT-PCR

Experiments were performed using the same samples extracted from the tumor tissues of age-matched PyMT and SB2−/−;PyMT mice for RNA-Seq. qRT-PCR was performed using an ABI PRISM 7900 system, SYBR Green PCR master mix (Applied Biosystems, Foster City, CA), and specific primer sets for Anxa3, Ccl17, Cxcl2, Cxcl13, Cxcr3, INF-γ, Itgad, Nr4a1 Sema3a, Tnfsf14, and Trem1 (Supplementary Table 4). The results were analyzed using the comparative ∆Ct method with the relative gene expression normalized to the γ-actin housekeeping gene [42].

### Statistical analysis

Statistical analyses were performed by GraphPad Prism 8.0 (GraphPad Software, Inc., La Jolla, CA). Statistical significance was determined by Student t-test to compare tumor size and number differences, and gene expression between two groups. For all tests, a *P*-value less than 0.05 was considered statistically significant.

## Supplementary Information

**Additional file 1: Supplementary Table 1.** 305 DEGs including upregulated and downregulated genes in SB2−/−;PyMT tumors.

**Additional file 2: Supplementary Table 2.** Subclasses of GO terms in the biological process (BP), molecular function (MF), and cellular compartment (CC) categories in SB2−/−;PyMT tumors compared to PyMT tumors.

**Additional file 3: Supplementary Table 3.** Detailed information of the DEGs associated with the functional categories regulated by SerpinB2 in SB2−/−;PyMT compared to PyMT.

**Additional file 4: Supplementary Table 4.** Specific primer sequences for qRT-PCR.

**Additional file 5: Supplementary Data 1.** Whole gel and membrane images for Fig. 1A, B.

## Data Availability

The raw transcriptome sequencing data (RNA-Seq) have been deposited in the BioProject under the accession number of PRJDB13249. The datasets supporting the conclusions of this article are included within the additional files. And any tumor samples and additional information about this study, are available from the corresponding authors on reasonable request.

## Abbreviations

ECM: Extracellular matrix
FC: Fold change
MMTV: Mouse mammary tumor virus
PyMT: Polyomavirus middle T antigen
SB2−/−;PyMT: SerpinB2-deficient PyMT
PAI-2: Plasminogen activator inhibitor-2
uPA: urokinase plasminogen activator
RNA-Seq: RNA Sequencing
DEGs: Differentially expressed genes
GO: Gene ontology
KEGG: Kyoto Encyclopedia of Genes and Genomes
