# Supplementary material for: Transcriptome analysis of SerpinB2-deficient breast tumors provides insight into deciphering SerpinB2-mediated roles in breast cancer progression
Source: BMC Genomics. 2022 Jun 29;23:479. doi: 10.1186/s12864-022-08704-4 (PMC9241327; doi:10.1186/s12864-022-08704-4)
Supplement: Supplementary file 1 — Additional file 1: Supplementary Table 1. 305 DEGs including upregulated and downregulated genes in SB2−/−;PyMT tumors. [file 12864_2022_8704_MOESM1_ESM.docx]

**Supplementary Table 1: 305 DEGs including upregulated and downregulated genes in SB2−/−;PyMT tumors.**

|  | **Filter: 305** |  | **Fold change** | ***p*-value** | **Average of normalized RC (log2)** |  |
| --- | --- | --- | --- | --- | --- | --- |
|  | **ID** | **Gene symbol** | **SB2-;-PyMT /PyMT** | **SB2-;-PyMT /PyMT** | **SB2-;-PyMT** | **PyMT** |
| **Up regulated genes** | 17061 | Smyd5 | 1.507 | 0.015 | 7.422 | 6.831 |
|  | 7497 | Gm16861 | 1.512 | 0.022 | 3.703 | 3.107 |
|  | 461 | Tm4sf20 | 1.512 | 0.026 | 0.861 | 0.264 |
|  | 562 | Fam132b | 1.515 | 0.032 | 7.155 | 6.556 |
|  | 14031 | Actl7b | 1.525 | 0.035 | 0.617 | 0.008 |
|  | 12563 | Slc17a9 | 1.547 | 0.016 | 4.972 | 4.342 |
|  | 2161 | Myrfl | 1.562 | 0.048 | 1.464 | 0.82 |
|  | 15498 | 0610040J01Rik | 1.569 | 0.016 | 9.63 | 8.98 |
|  | 15731 | Anxa3 | 1.58 | 0.008 | 9.97 | 9.31 |
|  | 16178 | Vps37d | 1.582 | 0.042 | 5.53 | 4.869 |
|  | 15778 | Gm17660 | 1.583 | 0.02 | 0.758 | 0.095 |
|  | 8263 | Airn | 1.587 | 0.03 | 5.235 | 4.569 |
|  | 8501 | Sox8 | 1.591 | 0.048 | 6.476 | 5.806 |
|  | 10295 | Trpm6 | 1.593 | 0.048 | 7.289 | 6.618 |
|  | 9874 | Hsbp1l1 | 1.64 | 0.026 | 5.256 | 4.543 |
|  | 814 | 5730559C18Rik | 1.64 | 0.007 | 5.993 | 5.279 |
|  | 22699 | Avpr2 | 1.641 | 0.044 | 3.398 | 2.683 |
|  | 12929 | Tmem144 | 1.645 | 0.031 | 4.842 | 4.124 |
|  | 16222 | Serpine1 | 1.658 | 0.025 | 7.895 | 7.165 |
|  | 5169 | Tubb2b | 1.665 | 0.015 | 11.745 | 11.01 |
|  | 12011 | Smox | 1.665 | 0.015 | 10.058 | 9.323 |
|  | 21905 | Faiml | 1.671 | 0.023 | 1.869 | 1.128 |
|  | 14192 | Gm13290 | 1.69 | 0.028 | 1.092 | 0.335 |
|  | 11315 | Zswim2 | 1.722 | 0.011 | 0.794 | 0.01 |
|  | 691 | Lypd1 | 1.789 | 0.038 | 1.504 | 0.665 |
|  | 5637 | Tmem171 | 1.79 | 0.047 | 1.914 | 1.074 |
|  | 20655 | Plekhg4 | 1.808 | 0.007 | 1.371 | 0.517 |
|  | 677 | 3110009E18Rik | 1.812 | 0.002 | 6.593 | 5.735 |
|  | 674 | Sctr | 1.822 | 0.024 | 3.085 | 2.219 |
|  | 8768 | Hspa1l | 1.827 | 0.023 | 3.836 | 2.966 |
|  | 9477 | Epb4.1l4a | 1.828 | 0.021 | 9.108 | 8.238 |
|  | 13776 | 4930480G23Rik | 1.83 | 0.033 | 0.885 | 0.013 |
|  | 6214 | Gm10364 | 1.833 | 0.035 | 2.645 | 1.771 |
|  | 4823 | Kif26a | 1.839 | 0.021 | 7.315 | 6.435 |
|  | 505 | Ecel1 | 1.847 | 0.02 | 1.269 | 0.384 |
|  | 21491 | Gm6981 | 1.853 | 0.039 | 1.935 | 1.046 |
|  | 9057 | Trem1 | 1.856 | 0.039 | 3.514 | 2.622 |
|  | 5571 | A830009L08Rik | 1.865 | 0.035 | 1.631 | 0.731 |
|  | 11336 | P2rx3 | 1.87 | 0.017 | 2.479 | 1.576 |
|  | 4419 | 4931403G20Rik | 1.9 | 0.04 | 1.356 | 0.43 |
|  | 17099 | D6Ertd527e | 1.902 | 0.008 | 5.04 | 4.113 |
|  | 23241 | Ddx3y | 1.943 | 0.04 | 1.121 | 0.163 |
|  | 15680 | Cxcl2 | 1.957 | 0.048 | 5.419 | 4.45 |
|  | 3944 | Rab37 | 1.964 | 0.049 | 5.531 | 4.557 |
|  | 20845 | Jph3 | 1.979 | 0.012 | 1.243 | 0.258 |
|  | 856 | Ptgs2 | 1.983 | 0.023 | 4.998 | 4.01 |
|  | 18466 | Zfp536 | 1.992 | 0.012 | 4.222 | 3.228 |
|  | 22453 | Zcchc12 | 2.002 | 0.05 | 1.016 | 0.015 |
|  | 7376 | Nr4a1 | 2.019 | 0.038 | 9.496 | 8.482 |
|  | 1209 | Hhipl2 | 2.062 | 0.043 | 8.147 | 7.103 |
|  | 14117 | Tnc | 2.099 | 0.02 | 9.34 | 8.27 |
|  | 17617 | Rerg | 2.111 | 0.029 | 6.432 | 5.355 |
|  | 8203 | Ldhal6b | 2.166 | 0.044 | 2.005 | 0.89 |
|  | 17281 | Mkrn2os | 2.17 | 0.025 | 2.119 | 1.002 |
|  | 6348 | Fam167a | 2.172 | 0.031 | 6.812 | 5.692 |
|  | 13538 | Col25a1 | 2.216 | 0.036 | 8.044 | 6.896 |
|  | 10577 | Gm19557 | 2.273 | 0.034 | 2.118 | 0.934 |
|  | 6227 | Fitm1 | 2.278 | 0.012 | 1.97 | 0.782 |
|  | 21150 | Tbx20 | 2.307 | 0.043 | 2.119 | 0.913 |
|  | 8963 | Adgrf1 | 2.32 | 0.033 | 3.72 | 2.506 |
|  | 5933 | Itih3 | 2.324 | 0.033 | 1.416 | 0.2 |
|  | 620 | Panct2 | 2.324 | 0.003 | 4.159 | 2.942 |
|  | 643 | Serpinb5 | 2.352 | 0 | 10.715 | 9.482 |
|  | 4143 | Dnmt3aos | 2.421 | 0.008 | 1.988 | 0.712 |
|  | 1378 | Slc35d3 | 2.457 | 0.05 | 5.276 | 3.979 |
|  | 17683 | 4930479D17Rik | 2.461 | 0.023 | 1.935 | 0.635 |
|  | 4010 | Snord1c | 2.482 | 0.016 | 1.823 | 0.511 |
|  | 10729 | St8sia6 | 2.661 | 0.005 | 7.35 | 5.937 |
|  | 16842 | Cpvl | 2.838 | 0.04 | 1.65 | 0.145 |
|  | 17492 | Clec2f | 2.909 | 0.049 | 4.518 | 2.978 |
|  | 2970 | Myocd | 2.992 | 0.031 | 2.134 | 0.553 |
|  | 690 | Gpr39 | 3.014 | 0.003 | 4.71 | 3.118 |
|  | 8993 | 1600014C23Rik | 3.378 | 0.046 | 3.133 | 1.377 |
|  | 650 | Serpinb11 | 4.758 | 0.006 | 6.016 | 3.766 |
|  | 654 | Serpinb8 | 4.771 | 0.016 | 8.529 | 6.275 |

| **Down regulated genes** | **Filter: 305** |  | **Fold change** | ***p*-value** | **Average of normalized RC (log2)** | |
| --- | --- | --- | --- | --- | --- | --- |
|  | **ID** | **Gene symbol** | **SB2-;-PyMT /PyMT** | **SB2-;-PyMT /PyMT** | **SB2-;-PyMT** | **PyMT** |
|  | 657 | Cdh19 | 0.089 | 0.033 | 1.532 | 5.023 |
|  | 15652 | Amtn | 0.178 | 0.022 | 5.303 | 7.791 |
|  | 14992 | Car6 | 0.253 | 0.014 | 9.71 | 11.691 |
|  | 19606 | Dmbt1 | 0.256 | 0.024 | 7.247 | 9.215 |
|  | 1274 | Syt14 | 0.27 | 0.01 | 0.468 | 2.358 |
|  | 17510 | Klre1 | 0.288 | 0.006 | 1.752 | 3.546 |
|  | 19845 | Retn | 0.289 | 0.048 | 5.079 | 6.87 |
|  | 13607 | Gbp2b | 0.297 | 0.032 | 0.599 | 2.351 |
|  | 8260 | Slc22a2 | 0.299 | 0.002 | 0.334 | 2.076 |
|  | 5546 | A830082K12Rik | 0.307 | 0.003 | 0.76 | 2.465 |
|  | 2068 | Epyc | 0.331 | 0.049 | 1.983 | 3.577 |
|  | 15727 | Cxcl13 | 0.332 | 0.034 | 3.734 | 5.325 |
|  | 14997 | 1700045H11Rik | 0.332 | 0.022 | 0.37 | 1.96 |
|  | 3927 | 4932435O22Rik | 0.346 | 0.03 | 0.146 | 1.678 |
|  | 4882 | Akr1c14 | 0.347 | 0.04 | 2.504 | 4.03 |
|  | 14217 | Elavl2 | 0.356 | 0.05 | 2.51 | 4.001 |
|  | 607 | D2hgdh | 0.363 | 0 | 2.998 | 4.462 |
|  | 16113 | Tmem132c | 0.363 | 0.035 | 2.608 | 4.068 |
|  | 15602 | Spink2 | 0.366 | 0.001 | 1.117 | 2.565 |
|  | 16624 | Akr1b7 | 0.366 | 0.027 | 0.971 | 2.419 |
|  | 6570 | Cldn10 | 0.381 | 0.04 | 5.995 | 7.385 |
|  | 9760 | Htr4 | 0.382 | 0.049 | 1.548 | 2.937 |
|  | 9812 | Mc2r | 0.386 | 0.007 | 0.855 | 2.229 |
|  | 13680 | AI606473 | 0.392 | 0.037 | 1.209 | 2.561 |
|  | 14108 | Orm2 | 0.402 | 0.006 | 1.031 | 2.344 |
|  | 1140 | Rgs7 | 0.404 | 0.006 | 0.698 | 2.006 |
|  | 21848 | Rasgrf1 | 0.405 | 0.021 | 3.856 | 5.16 |
|  | 16827 | Hoxa10 | 0.405 | 0.046 | 2.821 | 4.124 |
|  | 4826 | A530016L24Rik | 0.407 | 0.022 | 2.484 | 3.781 |
|  | 20577 | Ccl17 | 0.413 | 0.008 | 2.498 | 3.774 |
|  | 14153 | Frem1 | 0.413 | 0.041 | 4.935 | 6.211 |
|  | 12103 | Pax1 | 0.414 | 0.021 | 0.483 | 1.754 |
|  | 10736 | Cacnb2 | 0.418 | 0.006 | 0.841 | 2.101 |
|  | 13679 | Lhx8 | 0.418 | 0.01 | 0.452 | 1.71 |
|  | 5239 | Rbm24 | 0.418 | 0.036 | 3.887 | 5.144 |
|  | 4328 | Coch | 0.421 | 0.018 | 0.968 | 2.218 |
|  | 17277 | Syn2 | 0.421 | 0.029 | 3.819 | 5.067 |
|  | 4727 | Gsc | 0.424 | 0.023 | 1.194 | 2.433 |
|  | 14810 | Pla2g2d | 0.426 | 0.031 | 3.742 | 4.974 |
|  | 11187 | Scn3a | 0.426 | 0.006 | 3.089 | 4.319 |
|  | 21004 | Izumo1r | 0.428 | 0.008 | 1.381 | 2.604 |
|  | 3905 | Abca8a | 0.433 | 0.033 | 6.161 | 7.368 |
|  | 1302 | Ccdc170 | 0.436 | 0.037 | 1.277 | 2.474 |
|  | 17555 | A630073D07Rik | 0.437 | 0.022 | 0.158 | 1.353 |
|  | 19502 | Sult1a1 | 0.438 | 0.021 | 4.13 | 5.321 |
|  | 5826 | Lrrc3b | 0.438 | 0.012 | 0.034 | 1.225 |
|  | 16184 | Fkbp6 | 0.439 | 0.043 | 1.752 | 2.94 |
|  | 5786 | Gm3317 | 0.444 | 0.017 | 0.513 | 1.683 |
|  | 8731 | H2-Eb2 | 0.448 | 0.022 | 1.13 | 2.289 |
|  | 6601 | Nalcn | 0.449 | 0.012 | 1.83 | 2.986 |
|  | 17717 | Prkcg | 0.45 | 0.024 | 0.379 | 1.53 |
|  | 17532 | Klra7 | 0.452 | 0.009 | 2.53 | 3.675 |
|  | 16570 | Lep | 0.453 | 0.05 | 3.472 | 4.614 |
|  | 15172 | Sema3a | 0.455 | 0.004 | 3.262 | 4.398 |
|  | 15438 | Gm7854 | 0.455 | 0.027 | 0.54 | 1.675 |
|  | 21834 | Trim43a | 0.456 | 0.006 | 0.158 | 1.292 |
|  | 16599 | Cpa2 | 0.458 | 0.024 | 0.255 | 1.383 |
|  | 9695 | Prdm6 | 0.459 | 0.018 | 1.317 | 2.441 |
|  | 20817 | Wfdc1 | 0.46 | 0.007 | 0.913 | 2.032 |
|  | 9598 | Pcdhga11 | 0.46 | 0.042 | 1.797 | 2.916 |
|  | 692 | Nckap5 | 0.462 | 0.05 | 3.891 | 5.004 |
|  | 7705 | Crygs | 0.463 | 0.009 | 0.135 | 1.246 |
|  | 10839 | Gm996 | 0.465 | 0.045 | 1.307 | 2.411 |
|  | 1608 | Oit3 | 0.467 | 0.004 | 0.611 | 1.71 |
|  | 6636 | Slc1a3 | 0.468 | 0.028 | 5.214 | 6.309 |
|  | 6645 | Spef2 | 0.47 | 0.046 | 3.222 | 4.31 |
|  | 2642 | Adra1b | 0.472 | 0.014 | 1.878 | 2.962 |
|  | 5136 | Irf4 | 0.472 | 0.043 | 3.544 | 4.628 |
|  | 17531 | Klra9 | 0.473 | 0.016 | 1.576 | 2.656 |
|  | 17360 | Mug-ps1 | 0.474 | 0.043 | 0.027 | 1.105 |
|  | 19609 | 5430419D17Rik | 0.476 | 0.005 | 0.03 | 1.102 |
|  | 5271 | Omd | 0.477 | 0.02 | 4.248 | 5.317 |
|  | 9903 | Cd226 | 0.477 | 0.049 | 2.614 | 3.682 |
|  | 5959 | Galnt15 | 0.477 | 0.047 | 1.612 | 2.68 |
|  | 7865 | Popdc2 | 0.477 | 0.029 | 2.28 | 3.347 |
|  | 14843 | Padi2 | 0.48 | 0.005 | 8.709 | 9.769 |
|  | 15241 | Asb10 | 0.485 | 0.012 | 1.755 | 2.8 |
|  | 20049 | Adam5 | 0.485 | 0.028 | 0.248 | 1.291 |
|  | 15790 | Gbp8 | 0.487 | 0.018 | 3.803 | 4.841 |
|  | 9085 | Efhb | 0.488 | 0.037 | 0.7 | 1.734 |
|  | 20331 | Il12rb1 | 0.49 | 0.01 | 1.926 | 2.956 |
|  | 10323 | Pgm5 | 0.494 | 0.024 | 7.651 | 8.669 |
|  | 4244 | Colec11 | 0.494 | 0.004 | 1.545 | 2.563 |
|  | 18891 | Sh3gl3 | 0.494 | 0.018 | 1.439 | 2.456 |
|  | 15390 | Cpz | 0.495 | 0.019 | 2.62 | 3.633 |
|  | 17952 | Slc8a2 | 0.496 | 0.049 | 1.589 | 2.602 |
|  | 6902 | 2010109I03Rik | 0.497 | 0.035 | 1.203 | 2.212 |
|  | 12941 | Rbm46 | 0.498 | 0.036 | 2.412 | 3.417 |
|  | 1993 | Stab2 | 0.5 | 0.013 | 0.656 | 1.655 |
|  | 16538 | Cped1 | 0.501 | 0.046 | 5.383 | 6.381 |
|  | 12463 | Dok5 | 0.502 | 0.019 | 2.439 | 3.434 |
|  | 4693 | Unc79 | 0.502 | 0.028 | 2.075 | 3.068 |
|  | 272 | Aox3 | 0.505 | 0.024 | 0.7 | 1.687 |
|  | 1269 | Kcnh1 | 0.512 | 0.044 | 5.874 | 6.84 |
|  | 11038 | Hc | 0.512 | 0.047 | 5.916 | 6.882 |
|  | 6992 | Apol7e | 0.512 | 0.026 | 1.209 | 2.174 |
|  | 20190 | Enpp6 | 0.512 | 0.047 | 3.523 | 4.488 |
|  | 23182 | Nhs | 0.516 | 0.047 | 2.11 | 3.065 |
|  | 2867 | Obscn | 0.517 | 0.048 | 4.046 | 4.999 |
|  | 1233 | Spata17 | 0.518 | 0.048 | 0.36 | 1.308 |
|  | 242 | Dnah7a | 0.519 | 0.026 | 0.842 | 1.789 |
|  | 18997 | Omp | 0.519 | 0.021 | 1.939 | 2.886 |
|  | 14475 | Gm12866 | 0.519 | 0.008 | 1.338 | 2.283 |
|  | 16661 | 1700025N23Rik | 0.52 | 0.024 | 0.47 | 1.414 |
|  | 2067 | Kera | 0.52 | 0.046 | 0.269 | 1.212 |
|  | 22240 | Cxcr6 | 0.522 | 0.039 | 4.589 | 5.527 |
|  | 19582 | Itgad | 0.522 | 0.045 | 0.984 | 1.921 |
|  | 7813 | E130310I04Rik | 0.524 | 0.041 | 0.849 | 1.78 |
|  | 15409 | Ppp2r2c | 0.525 | 0.012 | 3.43 | 4.36 |
|  | 12856 | P2ry1 | 0.526 | 0.024 | 6.001 | 6.929 |
|  | 8891 | Olfr99 | 0.529 | 0.035 | 0.256 | 1.175 |
|  | 1279 | Hsd11b1 | 0.53 | 0.015 | 5.015 | 5.931 |
|  | 2800 | Ccdc69 | 0.53 | 0.015 | 3.806 | 4.72 |
|  | 10686 | Ccdc3 | 0.531 | 0.048 | 4.315 | 5.227 |
|  | 12167 | 6820408C15Rik | 0.531 | 0.027 | 0.729 | 1.642 |
|  | 5211 | Elovl2 | 0.534 | 0.036 | 1.158 | 2.064 |
|  | 4227 | Cys1 | 0.534 | 0.044 | 2.959 | 3.864 |
|  | 13204 | Gm128 | 0.535 | 0.015 | 2.304 | 3.207 |
|  | 20240 | Spock3 | 0.537 | 0.046 | 1.072 | 1.968 |
|  | 8624 | Dnah8 | 0.54 | 0.049 | 2.005 | 2.895 |
|  | 14939 | Gm13152 | 0.54 | 0.035 | 0.988 | 1.877 |
|  | 16410 | Flt3 | 0.541 | 0.012 | 2.544 | 3.43 |
|  | 21753 | 4933433G15Rik | 0.542 | 0.012 | 0.781 | 1.664 |
|  | 18403 | Mag | 0.543 | 0.027 | 2.423 | 3.305 |
|  | 18541 | Klk1b27 | 0.544 | 0.008 | 0.022 | 0.899 |
|  | 5724 | Ccno | 0.545 | 0.031 | 0.149 | 1.024 |
|  | 17160 | Klf15 | 0.546 | 0.035 | 3.181 | 4.055 |
|  | 8288 | Smoc2 | 0.549 | 0.049 | 8.68 | 9.545 |
|  | 18273 | Gm10046 | 0.551 | 0.014 | 1.033 | 1.894 |
|  | 18506 | Siglecg | 0.551 | 0.009 | 1.212 | 2.073 |
|  | 9725 | Gm4841 | 0.551 | 0.029 | 4.005 | 4.865 |
|  | 14618 | Zbtb8b | 0.551 | 0.002 | 3.732 | 4.591 |
|  | 4800 | Amn | 0.553 | 0.012 | 2.963 | 3.818 |
|  | 4720 | Serpina3f | 0.553 | 0.02 | 1.57 | 2.425 |
|  | 21197 | Ddx25 | 0.553 | 0.041 | 0.396 | 1.25 |
|  | 20179 | Lrp2bp | 0.554 | 0.014 | 1.856 | 2.707 |
|  | 3862 | Cd79b | 0.556 | 0.032 | 1.895 | 2.742 |
|  | 15707 | Ccdc158 | 0.559 | 0.033 | 2.711 | 3.549 |
|  | 16166 | Eln | 0.561 | 0.04 | 10.204 | 11.036 |
|  | 14772 | Htr1d | 0.561 | 0.036 | 3.771 | 4.604 |
|  | 7456 | Gpr84 | 0.563 | 0.027 | 0.574 | 1.404 |
|  | 22154 | Eomes | 0.564 | 0.04 | 3.717 | 4.542 |
|  | 8073 | Cldn17 | 0.564 | 0.038 | 0.018 | 0.843 |
|  | 17657 | Gm7457 | 0.565 | 0.014 | 0.263 | 1.088 |
|  | 21153 | Eepd1 | 0.565 | 0.001 | 5.056 | 5.879 |
|  | 1488 | Hs3st5 | 0.567 | 0.034 | 0.554 | 1.372 |
|  | 20827 | A330074K22Rik | 0.567 | 0.047 | 0.388 | 1.205 |
|  | 14272 | Gm12709 | 0.568 | 0.033 | 0.346 | 1.161 |
|  | 615 | St8sia4 | 0.571 | 0.025 | 6.266 | 7.074 |
|  | 271 | Aox1 | 0.573 | 0.001 | 4.286 | 5.089 |
|  | 3612 | Ikzf3 | 0.573 | 0.044 | 3.313 | 4.115 |
|  | 17740 | Pira11 | 0.574 | 0.019 | 1.029 | 1.829 |
|  | 2183 | Ifng | 0.575 | 0.038 | 0.349 | 1.148 |
|  | 22378 | Gpr34 | 0.575 | 0.045 | 5.107 | 5.905 |
|  | 5774 | D830030K20Rik | 0.576 | 0.045 | 0.739 | 1.535 |
|  | 11950 | Fbln7 | 0.576 | 0.037 | 6.814 | 7.609 |
|  | 1686 | Tmem26 | 0.576 | 0.036 | 2.622 | 3.417 |
|  | 15802 | Hfm1 | 0.578 | 0.02 | 1.227 | 2.018 |
|  | 20615 | Cmtm2a | 0.579 | 0.04 | 0.278 | 1.065 |
|  | 21347 | Clmp | 0.58 | 0.039 | 7.762 | 8.546 |
|  | 18812 | Gm10619 | 0.581 | 0.048 | 0.184 | 0.967 |
|  | 13548 | Dkk2 | 0.581 | 0.03 | 4.42 | 5.202 |
|  | 3826 | Wnt9b | 0.585 | 0.046 | 0.867 | 1.641 |
|  | 9505 | Slc23a1 | 0.586 | 0.02 | 3.326 | 4.099 |
|  | 20785 | Clec3a | 0.586 | 0.003 | 0.128 | 0.9 |
|  | 2160 | 4930579P08Rik | 0.586 | 0.038 | 0.863 | 1.634 |
|  | 20546 | Slc6a2 | 0.587 | 0.045 | 6.541 | 7.309 |
|  | 15471 | C130083M11Rik | 0.589 | 0.036 | 1.003 | 1.766 |
|  | 2895 | Med9os | 0.589 | 0.035 | 2.727 | 3.489 |
|  | 13563 | Bdh2 | 0.591 | 0.034 | 3.711 | 4.471 |
|  | 12628 | Fabp12 | 0.592 | 0.015 | 0.128 | 0.884 |
|  | 652 | Serpinb2 | 0.593 | 0.05 | 0.017 | 0.772 |
|  | 20719 | Zfhx3 | 0.596 | 0.038 | 9.818 | 10.564 |
|  | 294 | Gm973 | 0.597 | 0.041 | 4.477 | 5.222 |
|  | 22500 | Rhox8 | 0.6 | 0.044 | 0.968 | 1.706 |
|  | 4377 | Clec14a | 0.6 | 0.04 | 5.612 | 6.349 |
|  | 17315 | Rasgef1a | 0.6 | 0.045 | 2.719 | 3.456 |
|  | 9740 | Slc6a7 | 0.601 | 0.014 | 0.161 | 0.896 |
|  | 823 | Nr5a2 | 0.602 | 0.042 | 2.576 | 3.309 |
|  | 22210 | 1700048O20Rik | 0.602 | 0.043 | 1.016 | 1.749 |
|  | 873 | Colgalt2 | 0.602 | 0.047 | 5.18 | 5.911 |
|  | 23223 | Amelx | 0.602 | 0.025 | 0.122 | 0.853 |
|  | 22832 | Awat2 | 0.603 | 0.019 | 2.458 | 3.189 |
|  | 540 | Platr5 | 0.605 | 0.034 | 0.478 | 1.202 |
|  | 9939 | Ptprcap | 0.606 | 0.02 | 3.215 | 3.939 |
|  | 785 | Mgat4e | 0.607 | 0.013 | 0.017 | 0.739 |
|  | 20149 | B430010I23Rik | 0.607 | 0.006 | 0.144 | 0.865 |
|  | 22857 | Cxcr3 | 0.608 | 0.047 | 2.487 | 3.205 |
|  | 2617 | Slit3 | 0.61 | 0.019 | 4.698 | 5.412 |
|  | 1311 | Ipcef1 | 0.61 | 0.036 | 3.652 | 4.366 |
|  | 11138 | Cacnb4 | 0.612 | 0.01 | 5.814 | 6.523 |
|  | 16778 | Gimap8 | 0.612 | 0.017 | 4.754 | 5.461 |
|  | 15504 | C030018K13Rik | 0.613 | 0.03 | 0.017 | 0.722 |
|  | 13316 | Nhlh2 | 0.614 | 0.022 | 0.015 | 0.72 |
|  | 3594 | Plxdc1 | 0.614 | 0.012 | 3.127 | 3.83 |
|  | 7446 | Hoxc5 | 0.615 | 0.019 | 1.395 | 2.097 |
|  | 13263 | Ankrd35 | 0.616 | 0.03 | 2.904 | 3.603 |
|  | 20258 | Nat1 | 0.616 | 0.032 | 0.429 | 1.127 |
|  | 10410 | Ppp1r3c | 0.618 | 0.048 | 8.212 | 8.907 |
|  | 20586 | Drc7 | 0.619 | 0.023 | 0.143 | 0.834 |
|  | 5192 | Cage1 | 0.62 | 0.021 | 2.834 | 3.525 |
|  | 3794 | 2810433D01Rik | 0.623 | 0.003 | 3.713 | 4.397 |
|  | 3923 | Cpsf4l | 0.623 | 0.036 | 5.842 | 6.524 |
|  | 23098 | Gm15097 | 0.625 | 0.047 | 0.015 | 0.694 |
|  | 17184 | Adamts9 | 0.625 | 0.03 | 4.172 | 4.849 |
|  | 5795 | Gm3558 | 0.626 | 0.005 | 0.926 | 1.602 |
|  | 8166 | Erg | 0.627 | 0.039 | 4.089 | 4.763 |
|  | 3846 | Gm11651 | 0.627 | 0.037 | 0.017 | 0.69 |
|  | 21453 | Tmprss5 | 0.628 | 0.029 | 0.229 | 0.901 |
|  | 7903 | Ccdc80 | 0.629 | 0.049 | 8.775 | 9.443 |
|  | 9160 | Tnfsf14 | 0.63 | 0.043 | 1.504 | 2.171 |
|  | 13927 | Fam221b | 0.631 | 0.015 | 0.128 | 0.791 |
|  | 17220 | Gm19757 | 0.633 | 0.031 | 0.015 | 0.674 |
|  | 3554 | Skap1 | 0.635 | 0.043 | 3.539 | 4.194 |
|  | 7669 | Cyp2ab1 | 0.636 | 0.039 | 0.126 | 0.778 |
|  | 6919 | Naprt | 0.641 | 0.042 | 3.541 | 4.183 |
|  | 16783 | Gimap1 | 0.642 | 0.038 | 5.186 | 5.826 |
|  | 15954 | Pla2g1b | 0.644 | 0.046 | 0.284 | 0.918 |
|  | 12903 | B3galnt1 | 0.646 | 0.048 | 5.127 | 5.758 |
|  | 15558 | Txk | 0.646 | 0.049 | 2.689 | 3.319 |
|  | 12306 | Arhgap40 | 0.647 | 0.046 | 0.653 | 1.281 |
|  | 3151 | Itgae | 0.65 | 0.044 | 3.207 | 3.828 |
|  | 15077 | Mxra8 | 0.65 | 0.038 | 9.473 | 10.094 |
|  | 9032 | Trerf1 | 0.65 | 0.041 | 3.614 | 4.235 |
|  | 10287 | Prune2 | 0.653 | 0.028 | 5.379 | 5.994 |
|  | 17456 | Dyrk4 | 0.653 | 0.039 | 0.013 | 0.627 |
|  | 5264 | Susd3 | 0.656 | 0.045 | 5.443 | 6.051 |
|  | 23210 | Trappc2 | 0.658 | 0.042 | 3.105 | 3.709 |
|  | 15194 | Magi2 | 0.662 | 0.041 | 4.242 | 4.836 |
|  | 21410 | Gm10684 | 0.665 | 0.047 | 5.209 | 5.798 |
|  | 856 | Ptgs2 | 1.983 | 0.023 | 4.998 | 4.01 |
|  | 18466 | Zfp536 | 1.992 | 0.012 | 4.222 | 3.228 |
|  | 22453 | Zcchc12 | 2.002 | 0.05 | 1.016 | 0.015 |
|  | 7376 | Nr4a1 | 2.019 | 0.038 | 9.496 | 8.482 |
|  | 1209 | Hhipl2 | 2.062 | 0.043 | 8.147 | 7.103 |
|  | 14117 | Tnc | 2.099 | 0.02 | 9.34 | 8.27 |
|  | 17617 | Rerg | 2.111 | 0.029 | 6.432 | 5.355 |
|  | 8203 | Ldhal6b | 2.166 | 0.044 | 2.005 | 0.89 |
|  | 17281 | Mkrn2os | 2.17 | 0.025 | 2.119 | 1.002 |
|  | 6348 | Fam167a | 2.172 | 0.031 | 6.812 | 5.692 |
|  | 13538 | Col25a1 | 2.216 | 0.036 | 8.044 | 6.896 |
|  | 10577 | Gm19557 | 2.273 | 0.034 | 2.118 | 0.934 |
|  | 6227 | Fitm1 | 2.278 | 0.012 | 1.97 | 0.782 |
|  | 21150 | Tbx20 | 2.307 | 0.043 | 2.119 | 0.913 |
|  | 8963 | Adgrf1 | 2.32 | 0.033 | 3.72 | 2.506 |
|  | 5933 | Itih3 | 2.324 | 0.033 | 1.416 | 0.2 |
|  | 620 | Panct2 | 2.324 | 0.003 | 4.159 | 2.942 |
|  | 643 | Serpinb5 | 2.352 | 0 | 10.715 | 9.482 |
|  | 4143 | Dnmt3aos | 2.421 | 0.008 | 1.988 | 0.712 |
|  | 1378 | Slc35d3 | 2.457 | 0.05 | 5.276 | 3.979 |
|  | 17683 | 4930479D17Rik | 2.461 | 0.023 | 1.935 | 0.635 |
|  | 4010 | Snord1c | 2.482 | 0.016 | 1.823 | 0.511 |
|  | 10729 | St8sia6 | 2.661 | 0.005 | 7.35 | 5.937 |
|  | 16842 | Cpvl | 2.838 | 0.04 | 1.65 | 0.145 |
|  | 17492 | Clec2f | 2.909 | 0.049 | 4.518 | 2.978 |
|  | 2970 | Myocd | 2.992 | 0.031 | 2.134 | 0.553 |
|  | 690 | Gpr39 | 3.014 | 0.003 | 4.71 | 3.118 |
|  | 8993 | 1600014C23Rik | 3.378 | 0.046 | 3.133 | 1.377 |
|  | 650 | Serpinb11 | 4.758 | 0.006 | 6.016 | 3.766 |
|  | 654 | Serpinb8 | 4.771 | 0.016 | 8.529 | 6.275 |
