# Supplementary material for: Transcriptome analysis of SerpinB2-deficient breast tumors provides insight into deciphering SerpinB2-mediated roles in breast cancer progression
Source: BMC Genomics. 2022 Jun 29;23:479. doi: 10.1186/s12864-022-08704-4 (PMC9241327; doi:10.1186/s12864-022-08704-4)
Supplement: Supplementary file 2 — Additional file 2: Supplementary Table 2. Subclasses of GO terms in the biological process (BP), molecular function (MF), and cellular compartment (CC) categories in SB2−/−;PyMT tumors compared to PyMT tumors. [file 12864_2022_8704_MOESM2_ESM.docx]

**Supplementary Table 2: Subclasses of GO terms in the biological process (BP), molecular function (MF), and cellular compartment (CC) categories in SB2−/−;PyMT tumors compared to PyMT tumors.**

| **Category** | **Term** |
| --- | --- |
| GO term-BP | GO:0010466~negative regulation of peptidase activity |
|  | GO:0007155~cell adhesion |
|  | GO:0032729~positive regulation of interferon-gamma production |
|  | GO:0032609~interferon-gamma production |
|  | GO:0006935~chemotaxis |
|  | GO:0042127~regulation of cell proliferation |
|  | GO:0070588~calcium ion transmembrane transport |
|  | GO:0002302~CD8-positive, alpha-beta T cell differentiation involved in immune response |
|  | GO:0050852~T cell receptor signaling pathway |
|  | GO:0002250~adaptive immune response |
|  | GO:0045987~positive regulation of smooth muscle contraction |
|  | GO:0007165~signal transduction |
|  | GO:0001560~regulation of cell growth by extracellular stimulus |
|  | GO:0007200~phospholipase C-activating G-protein coupled receptor signaling pathway |
|  | GO:0009612~response to mechanical stimulus |
|  | GO:0042475~odontogenesis of dentin-containing tooth |
|  | GO:0035589~G-protein coupled purinergic nucleotide receptor signaling pathway |
|  | GO:0071346~cellular response to interferon-gamma |
|  | GO:0042832~defense response to protozoan |
|  | GO:0030593~neutrophil chemotaxis |
|  | GO:0009115~xanthine catabolic process |
|  | GO:0070100~negative regulation of chemokine-mediated signaling pathway |
|  | GO:0007268~chemical synaptic transmission |
|  | GO:0051384~response to glucocorticoid |
|  | GO:0060385~axonogenesis involved in innervation |
|  | GO:0030183~B cell differentiation |
|  | GO:0007528~neuromuscular junction development |
|  | GO:1901385~regulation of voltage-gated calcium channel activity |
|  | GO:0043270~positive regulation of ion transport |
|  | GO:0007275~multicellular organism development |
|  | GO:0030217~T cell differentiation |
|  | GO:0010042~response to manganese ion |
|  | GO:0006816~calcium ion transport |
|  | GO:0045893~positive regulation of transcription, DNA-templated |
| GO term-MF | GO:0004867~serine-type endopeptidase inhibitor activity |
|  | GO:0030414~peptidase inhibitor activity |
|  | GO:0030246~carbohydrate binding |
|  | GO:0008201~heparin binding |
|  | GO:0016614~oxidoreductase activity, acting on CH-OH group of donors |
|  | GO:0004871~signal transducer activity |
|  | GO:0005044~scavenger receptor activity |
|  | GO:0005539~glycosaminoglycan binding |
|  | GO:0019958~C-X-C chemokine binding |
|  | GO:0005125~cytokine activity |
|  | GO:0001077~transcriptional activator activity, RNA polymerase II core promoter proximal region sequence-specific binding |
|  | GO:0005509~calcium ion binding |
|  | GO:0004854~xanthine dehydrogenase activity |
|  | GO:0004031~aldehyde oxidase activity |
|  | GO:0004180~carboxypeptidase activity |
|  | GO:0001102~RNA polymerase II activating transcription factor binding |
|  | GO:0016494~C-X-C chemokine receptor activity |
|  | GO:0043565~sequence-specific DNA binding |
| GO term-CC | GO:0005576~extracellular region |
|  | GO:0005578~proteinaceous extracellular matrix |
|  | GO:0005615~extracellular space |
|  | GO:0009897~external side of plasma membrane |
|  | GO:0016020~membrane |
|  | GO:0005614~interstitial matrix |
|  | GO:0005604~basement membrane |
|  | GO:0009986~cell surface |
|  | GO:0005923~bicellular tight junction |
