# Supplementary material for: Transcriptome analysis of SerpinB2-deficient breast tumors provides insight into deciphering SerpinB2-mediated roles in breast cancer progression
Source: BMC Genomics. 2022 Jun 29;23:479. doi: 10.1186/s12864-022-08704-4 (PMC9241327; doi:10.1186/s12864-022-08704-4)
Supplement: Supplementary file 3 — Additional file 3: Supplementary Table 3. Detailed information of the DEGs associated with the functional categories regulated by SerpinB2 in SB2−/−;PyMT compared to PyMT. [file 12864_2022_8704_MOESM3_ESM.docx]

**Supplementary Table 3: Detailed information of the DEGs associated with the functional categories regulated by SerpinB2 in SB2−/−;PyMT compared to PyMT.**

| **Functional**  **categories** | **Gene symbol** | **Description** | **Fold**  **change** | ***P*-value** |
| --- | --- | --- | --- | --- |
| Cell death | Cxcl2 | Chemokine (C-X-C motif) ligand 2 | 1.957 | 0.048 |
|  | Nr4a1 | Nuclear receptor subfamily 4, group A, member 1 | 2.019 | 0.038 |
|  | Zswim2 | Zinc finger SWIM-type containing 2 | 1.722 | 0.011 |
|  | Prune2 | Prune homolog 2 (Drosophila) | 0.653 | 0.028 |
|  | IFN-γ | Interferon gamma | 0.575 | 0.038 |
|  | Tnfsf14 | Tumor necrosis factor (ligand) superfamily, member 14 | 0.63 | 0.043 |
|  | Sema3a | Sema domain, immunoglobulin domain (Ig), short basic domain,secreted, (semaphorin) 3A | 0.455 | 0.004 |
| Cell proliferation | Pax1  Itgad  Flt3  Spink2 | Paired box 1 | 0.414 | 0.021 |
|  |  | Integrin, alpha D | 0.522 | 0.045 |
|  |  | FMS-like tyrosine kinase 3 | 0.541 | 0.012 |
|  |  | Serine peptidase inhibitor, Kazal type 2 | 0.366 | 0.001 |
| Cell cycle | Zfhx3  Magi2  Ccno  IFN-γ  Ccdc69  Hfm1  Fkbp6 | Zinc finger homeobox 3 | 0.596 | 0.038 |
|  |  | Membrane associated guanylate kinase, WW and PDZ domain containing 2 | 0.662 | 0.041 |
|  |  | Cyclin O | 0.545 | 0.031 |
|  |  | Interferon gamma | 0.575 | 0.038 |
|  |  | Coiled-coil domain containing 69 | 0.53 | 0.015 |
|  |  | HFM1, ATP-dependent DNA helicase homolog | 0.578 | 0.02 |
|  |  | FK506 binding protein 6 | 0.439 | 0.043 |
| Immune  response | 5730559C18Rik | RIKEN cDNA 5730559C18 gene | 1.64 | 0.007 |
|  | Cxcl2 | Chemokine (C-X-C motif) ligand 2 | 1.957 | 0.048 |
|  | Trem1 | Triggering receptor expressed on myeloid cells 1 | 1.856 | 0.039 |
|  | Anxa3 | Annexin A3 | 1.58 | 0.008 |
|  | Il12rb1 | Interleukin 12 receptor, beta 1 | 0.49 | 0.01 |
|  | Pla2g1b | Phospholipase A2, group IB, pancreas | 0.644 | 0.046 |
|  | IFN-γ | Interferon gamma | 0.575 | 0.038 |
|  | Eomes | Eomesodermin | 0.564 | 0.04 |
|  | Cd79b | CD79B antigen | 0.556 | 0.032 |
|  | Txk | TXK tyrosine kinase | 0.646 | 0.049 |
|  | Irf4 | Interferon regulatory factor 4 | 0.472 | 0.043 |
|  | H2-Eb2 | Histocompatibility 2, class II antigen E beta2 | 0.448 | 0.022 |
|  | Tnfsf14 | Tumor necrosis factor (ligand) superfamily, member 14 | 0.63 | 0.043 |
|  | Gbp8 | Guanylate-binding protein 8 | 0.487 | 0.018 |
|  | Ccl17 | Chemokine (C-C motif) ligand 17 | 0.413 | 0.008 |
|  | Cxcl13 | Chemokine (C-X-C motif) ligand 13 | 0.332 | 0.034 |
| Inflammatory  response | Ptgs2 | Prostaglandin-endoperoxide synthase 2 | 1.983 | 0.023 |
|  | Cxcl2 | Chemokine (C-X-C motif) ligand 2 | 1.957 | 0.048 |
|  | Cxcr3 | Chemokine (C-X-C motif) receptor 3 | 0.608 | 0.047 |
| Cell adhesion | Cldn17 | Claudin 17 | 0.564 | 0.038 |
|  | Izumo1r | IZUMO1 receptor, JUNO | 0.428 | 0.008 |
|  | Itgad | Integrin alpha E, epithelial-associated | 0.522 | 0.045 |
|  | Amelx | Amelogenin, X-linked | 0.602 | 0.025 |
|  | Cdh19 | Cadherin 19, type 2 | 0.089 | 0.033 |
|  | Stab2 | Stabilin 2 | 0.5 | 0.013 |
|  | Klra7 | Killer cell lectin-like receptor, subfamily A, member 7 | 0.452 | 0.009 |
|  | Pcdhga11 | Protocadherin gamma subfamily A, 11 | 0.46 | 0.042 |
|  | Adamts9 | A disintegrin-like and metallopeptidase (reprolysin type) with thrombospondin type 1 motif, 9 | 0.625 | 0.03 |
|  | Amtn | Amelotin | 0.178 | 0.022 |
|  | Omd | Osteomodulin | 0.477 | 0.02 |
|  | Frem1 | Fras1 related extracellular matrix protein 1 | 0.413 | 0.041 |
|  | Lep | Leptin | 0.453 | 0.05 |
|  | Cd226 | CD226 antigen | 0.477 | 0.049 |
|  | Itgad | Integrin, alpha D | 0.522 | 0.045 |
|  | Cldn10 | Claudin 10 | 0.381 | 0.04 |
|  | Mag | Myelin-associated glycoprotein | 0.543 | 0.027 |
|  | Fbln7 | Fibulin 7 | 0.576 | 0.037 |
|  | Pgm5 | Phosphoglucomutase 5 | 0.494 | 0.024 |
|  | Mxra8 | Matrix-remodelling associated 8 | 0.65 | 0.038 |
|  | Tnc | Tenascin C | 2.099 | 0.02 |
|  | Anxa3 | Annexin A3 | 1.58 | 0.008 |
|  | Serpinb8 | Serine (or cysteine) peptidase inhibitor, clade B, member 8 | 4.771 | 0.016 |
| Cell migration | Trem1 | Triggering receptor expressed on myeloid cells 1 | 1.856 | 0.039 |
|  | Tubb2b | Tubulin, beta 2B class IIB | 1.665 | 0.015 |
|  | Nr4a1 | Nuclear receptor subfamily 4, group A, member 1 | 2.019 | 0.038 |
|  | Cxcl2 | Chemokine (C-X-C motif) ligand 2 | 1.957 | 0.048 |
|  | Sox8 | SRY (sex determining region Y)-box 8 | 1.591 | 0.048 |
|  | IFN-γ | Interferon gamma | 0.575 | 0.038 |
|  | Cxcr3 | Chemokine (C-X-C motif) receptor 3 | 0.608 | 0.047 |
|  | Rasgef1a | RasGEF domain family, member 1A | 0.6 | 0.045 |
|  | Erg | Avian erythroblastosis virus E-26 (v-ets) oncogene related | 0.627 | 0.039 |
|  | Sema3a | Sema domain, immunoglobulin domain (Ig), short basic domain, secreted, (semaphorin) 3A | 0.455 | 0.004 |
|  | P2ry1 | Purinergic receptor P2Y, G-protein coupled 1 | 0.526 | 0.024 |
|  | Lep | Leptin | 0.453 | 0.05 |
|  | Ccl17 | Chemokine (C-C motif) ligand 17 | 0.413 | 0.008 |
|  | Cxcl13 | Chemokine (C-X-C motif) ligand 13 | 0.332 | 0.034 |
| Extracellualr  matrix | Tnc | Tenascin C | 2.099 | 0.02 |
|  | Serpine1 | Serine (or cysteine) peptidase inhibitor,  Clade E, member 1 | 1.658 | 0.025 |
|  | Amelx | Amelogenin, X-linked | 0.625 | 0.03 |
|  | Wnt9b | Wingless-type MMTV integration site family,  Member 9B | 0.585 | 0.046 |
|  | Adamts9 | A disintegrin-like and metallopeptidase (reprolysin type) with thrombospondin type 1 motif, 9 | 0.625 | 0.03 |
|  | Spock3 | Sparc/osteonectin, cwcv and kazal-like domains proteoglycan 3 | 0.537 | 0.046 |
|  | Coch | Cochlin | 0.421 | 0.018 |
|  | Kera | Keratocan | 0.52 | 0.046 |
|  | Omd | Osteomodulin | 0.477 | 0.02 |
|  | Epyc | Epiphycan | 0.331 | 0.049 |
|  | Fbln7 | Fibulin 7 | 0.576 | 0.037 |
|  | Cpz | Carboxypeptidase Z | 0.495 | 0.019 |
|  | Clec14a | C-type lectin domain family 14, member a | 0.6 | 0.04 |
|  | Ccdc80 | Coiled-coil domain containing 80 | 0.629 | 0.049 |
|  | Smoc2 | SPARC related modular calcium binding 2 | 0.549 | 0.049 |
|  | Frem1 | Fras1 related extracellular matrix protein 1 | 0.413 | 0.041 |
|  | Eln | Elastin | 0.561 | 0.04 |
|  | Amtn | Amelotin | 0.178 | 0.022 |
| Wound healing | Ptgs2 | Purinergic receptor P2Y, G-protein coupled 1 | 1.983 | 0.023 |
|  | Serpinb2 | Serine (or cysteine) peptidase inhibitor, clade B, member 2 | 0.593 | 0.05 |
|  | Cxcl2 | Chemokine (C-X-C motif) ligand 2 | 1.957 | 0.048 |
|  | Cxcr3 | Tenascin C | 0.608 | 0.047 |
