# Supplementary material for: Transcriptome analysis of SerpinB2-deficient breast tumors provides insight into deciphering SerpinB2-mediated roles in breast cancer progression
Source: BMC Genomics. 2022 Jun 29;23:479. doi: 10.1186/s12864-022-08704-4 (PMC9241327; doi:10.1186/s12864-022-08704-4)
Supplement: Supplementary file 4 — Additional file 4: Supplementary Table 4. Specific primer sequences for qRT-PCR. [file 12864_2022_8704_MOESM4_ESM.docx]

**Supplementary Table 4. Specific primer sequences for qRT-PCR.**

| **Gene** | **Sequence (5′**$\boldsymbol{->}$**3′)** | |
| --- | --- | --- |
| Anxa3 | Forward | GAACAAATGGGGCACAGACG |
|  | Reverse | TGGAGTGTTCCTCGCACAAT |
| Ccl17 | Forward | GGCCGAGAGTGCTGCCTGGA |
|  | Reverse | GCCCTGGACAGTCAGAAACACGA |
| Cxcl2 | Forward | CCCTGCCAAGGGTTGACTT |
|  | Reverse | GGCAAACTTTTTGACCGCCC |
| Cxcl13 | Forward | CGGATTCAAGTTACGCCCCC |
|  | Reverse | GCTTGGGGAGTTGAAGACAGA |
| Cxcr3 | Forward | AATGCCACCCATTGCCAGTA |
|  | Reverse | TAGCTCGAAAACGCCTCTGG |
| INF-γ | Forward | ACGGCACAGTCATTGAAAGC |
|  | Reverse | TGCTGATGGCCTGATTGTCTT |
| Itgad | Forward | CCTCGTTCTCCCTGATGCAA |
|  | Reverse | GCCTGTTGCTGTCTACCACT |
| Nr4a1 | Forward | CGGCCCATTAGATGAGACCC |
|  | Reverse | TCATAAGTCTGGCTCGGGGA |
| Sema3a | Forward | TGCAGCTCATTAACCACCCC |
|  | Reverse | ACTTGTTGCTGCTCCCTTGA |
| Tnfsf14 | Forward | AGCCAACGCCAGCTTGATAG |
|  | Reverse | CATGATACGTCAAGCCCCTCA |
| Trem1 | Forward | ACTGCTGTGCGTGTTCTTTG |
|  | Reverse | TGGGTAGGGATCGGGTTGTA |
| γ-actin | Forward | TTCCTGGGCATGGAGTCCTGTGG |
|  | Reverse | CGCCTAGAAGCATTTGCGGTGG |
