# Supplementary material for: Transcriptome analysis of SerpinB2-deficient breast tumors provides insight into deciphering SerpinB2-mediated roles in breast cancer progression
Source: BMC Genomics. 2022 Jun 29;23:479. doi: 10.1186/s12864-022-08704-4 (PMC9241327; doi:10.1186/s12864-022-08704-4)
Supplement: Supplementary file 5 — Additional file 5: Supplementary Data 1. Whole gel and membrane images for Fig. 1A, B. [file 12864_2022_8704_MOESM5_ESM.docx]

**Supplementary Data 1: Whole gel and membrane images for Figure 1A, 1B.**


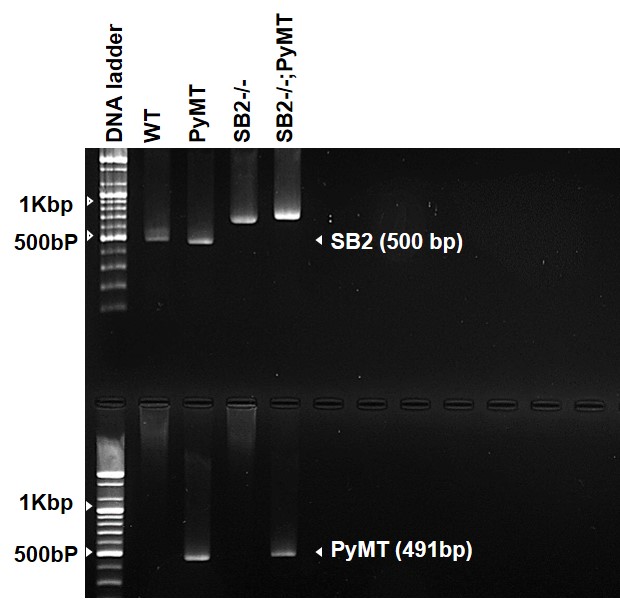


**(A) Full-length image for Figure 1A.**


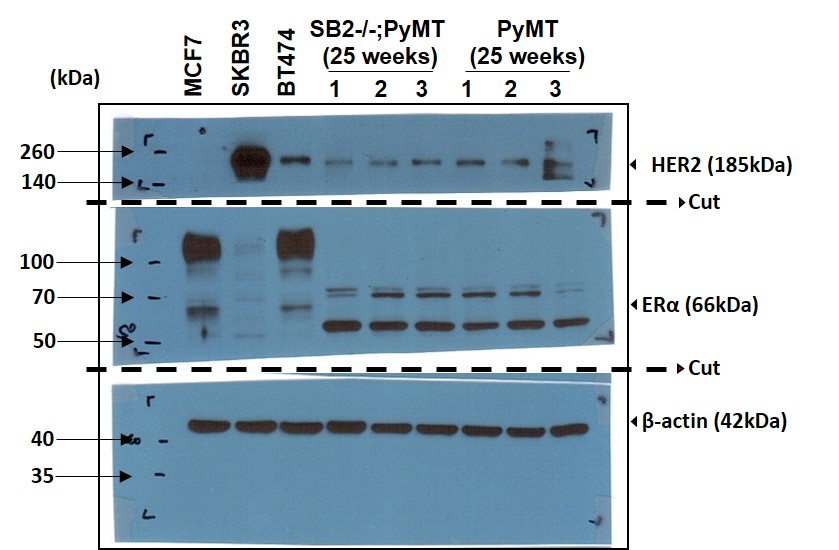


**(B) Raw data for Figure 1B.** The whole membrane was cut into three pieces prior to hybridization with different primary antibodies. The first strip was stained with HER2 antibody (185 kDa), the second strip was stained with ERa antibody (66kDa), and the third strip was stained with β-actin (42kDa).
